# Impacts of COVID‐19 on early childhood mental health

**DOI:** 10.1002/imhj.70005

**Published:** 2025-02-20

**Authors:** Marissa Marsolek, Kathy Dowell

**Affiliations:** ^1^ Department of Psychology University of Minnesota Duluth Duluth Minnesota USA

**Keywords:** caregiver‐child relationship, COVID‐19, early childhood mental health, externalizing behaviors, mental health services, **الكلمات المفتاحية**: الإشراف التأملي، الممارسة التأملية، الزيارة المنزلية، الرعاية والتعليم المبكر، التنفيذ, COVID‐19, 幼儿心理健康, 看护者‐儿童关系, 外化行为, COVID‐19, santé mentale des jeunes enfants, relation enfant‐aidant, comportements externalisants, COVID‐19, frühkindliche psychische Gesundheit, Eltern‐Kind‐Beziehung, externalisierende Verhaltensweisen, COVID‐19, 幼児期のメンタルヘルス, 養育者と子どもの関係性, 外在化行動, COVID‐19, salud mental en la temprana niñez, relación cuidador‐niño, comportamientos externalizantes

## Abstract

This study investigates the impact of the COVID‐19 pandemic on young children's mental health and their engagement in mental health services in the midwestern United States. Previous research investigating the impact of COVID‐19 on young children's mental health service utilization has rarely included children under 5 years. Therefore, this study examined how the COVID‐19 pandemic and the caregiver‐child relationship impacted children's mental health symptoms. It also investigated the impact of COVID‐19 and the child's symptom presentation on caregivers’ engagement and attendance in mental health treatment. Data were collected with children 0 to 5 years old (*n* = 486) from January 2017 to April 2022 using archival records from a community mental health organization. Participants were primarily low‐income (81.9%) and White (81.3%). Results found that the caregiver‐child relationship impacted children's mental health symptoms before and during the COVID‐19 pandemic. Both caregivers and children were rated as more engaged after the pandemic began if the child experienced externalizing symptoms. Children attended fewer therapy sessions after the pandemic started, and those with externalizing symptoms received more overall services than those without.

## ملخص

يُنظر إلى الإشراف التأملي(RS) على أنه يندرج تحت أفضل الممارسات، وبالتالي يتم دمجه—وغالبًا ما يتم فرضه—كميزة رئيسية للعديد من برامج خدمة الرضع والطفولة المبكرة القائمة على العلاقات. ولتعزيز تنفيذ الإشراف التأملي عالي الجودة للمهنيين العاملين في مجال الرضع والطفولة المبكرة، من المهم التركيز على كيفية تدريب المهنيين العاملين في مجال الرضع والطفولة المبكرة على بناء قدرات الإشراف التأملي. تحقيقًا لهذه الغاية، نصف رحلة إحدى الولايات الأمريكية في تطوير وتنفيذ وتطويع سلسلة تطوير امهني في مجال العلاقات والتواصل مع الأطفال الرضع والطفولة المبكرة. نصف هيكل المناهج الدراسية بالإضافة إلى المحتوى والأهداف التعليمية، والتي تسعى إلى سد الفجوة بين المفاهيم النظرية الأساسية لمهارات واستراتيجيات دعم العلاقات مع الطفل، والتأمل الذاتي الموجه نحو العمليات، والتطبيق العملي لمهارات واستراتيجيات دعم العلاقات مع الطفل. كما نوجز أيضًا عملية التطوير والتطويع التكراري التي صقلت المناهج الدراسية على مدار العقد الماضي. وأخيراً ، نعرض موجزاً لتاريخ التنسيق والتعاون الذي عزز تطوير وتنفيذ هذه السلسلة التي تم نشرها في إطار الزيارات المنزلية وبيئات الرعاية والتعليم المبكر. يمكن أن يكون هذا السرد بمثابة نموذج للمنظمات والأنظمة والولايات التي تبذل جهودًا لتوفير التطوير المهني الذي يركز على الإشراف التأملي.

## 摘 要

这项研究探讨了COVID‐19疫情对美国中西部地区幼儿心理健康及其参与心理健康服务的影响。此前针对COVID‐19疫情对幼儿心理健康服务利用的研究很少涵盖5岁以下的儿童。因此, 本研究考察了COVID‐19疫情及看护者‐儿童关系对儿童心理健康症状的影响, 同时还研究了COVID‐19疫情和儿童症状表现对看护者参与和出席心理健康治疗的影响。数据来自一个社区心理健康机构的档案记录, 从2017年1月到2022年4月间收集了0至5岁儿童(*n* = 486)的相关数据。参与者主要为低收入家庭(81.9%)和白人(81.3%)。研究结果显示, 看护者‐儿童关系在COVID‐19疫情前后均对儿童的心理健康症状产生了影响。如果儿童表现出外化症状, 则疫情开始后看护者和儿童的参与度评分更高。然而, 儿童整体参与治疗的次数在疫情开始后有所减少, 同时表现出外化症状的儿童接受的总服务次数多于未表现出外化症状的儿童。

## Résumé

Cette étude porte sur l'impact sur la santé mentale des jeunes enfants de la pandémie du COVID‐19 et ainsi que sur leur participation dans les services de santé mentale dans la région américaine du Midwest (Etats‐Unis). Jusqu’à présent les recherches portant sur l'impact du COVID‐19 sur l'utilisation des services de santé mentale par les jeunes enfants ont rarement inclus les enfants de moins de 5 ans. Par conséquent, cette étude a examiné la façon dont la pandémie du COVID‐19 et la relation entre la personne prenant soin de l'enfant et l'enfant ont affecté les symptômes de santé mentale des enfants. Notre étude s'est également penchée sur l'impact du COVID‐19 et la présentation des symptômes par l'enfant sur la participation et la présence des personnes prenant soin de l'enfant dans le traitement en santé mentale. Les données ont été recueillies auprès d'enfants âgés de 0 à 5 ans (*n* = 486) de janvier 2017 à avril 2022, au moyen de dossiers archivés provenant d'un centre communautaire de santé mentale. Les participants étaient principalement des personnes à faible revenu (81,9 %) et des blancs (81,3 %). Les résultats ont révélé que la relation entre la personne prenant soin de l'enfant et l'enfant a eu une incidence sur les symptômes de santé mentale des enfants avant et pendant la pandémie du COVID‐19. Les personnes s'occupant des enfants et les enfants ont tous été évalués comme étant plus engagés après le début de la pandémie si l'enfant présentait des symptômes d'externalisation. Les enfants ont assisté à moins de séances de thérapie après le début de la pandémie, et ceux qui présentaient des symptômes d'externalisation ont reçu plus de services que ceux qui n'en présentaient pas.

## Zusammenfassung

Diese Studie untersucht die Auswirkungen der COVID‐19‐Pandemie auf die psychische Gesundheit von Kleinkindern und ihre Inanspruchnahme von psychotherapeutischen Dienstleistungen im mittleren Westen der USA. Bisherige Untersuchungen zu den Auswirkungen der COVID‐19‐Pandemie auf die Inanspruchnahme solcher Dienste durch Kleinkinder haben selten Kinder unter 5 Jahren einbezogen. Daher wurde in dieser Studie untersucht, wie sich die COVID‐19‐Pandemie und die Beziehung zwischen Bezugsperson und Kind auf die psychischen Symptome der Kinder auswirken. Außerdem wurde untersucht, wie sich COVID‐19 und die Symptomdarstellung des Kindes auf das Engagement der Bezugspersonen und die Teilnahme an psychotherapeutischen Behandlungen auswirken. Die Daten wurden mit Kindern im Alter von 0 bis 5 Jahren (*n* = 486) von Januar 2017 bis April 2022 anhand von Archivaufzeichnungen einer kommunalen Einrichtung für psychische Gesundheit erhoben. Die Teilnehmer waren überwiegend einkommensschwach (81,9 %) und weiß (81,3 %). Die Ergebnisse zeigten, dass sich die Beziehung zwischen Bezugsperson und Kind auf die psychischen Symptome der Kinder vor und während der COVID‐19‐Pandemie auswirkte. Sowohl die Bezugspersonen als auch die Kinder wurden nach Beginn der Pandemie als engagierter eingestuft, wenn das Kind externalisierende Symptome aufwies. Kinder nahmen nach Beginn der Pandemie weniger Therapiesitzungen wahr, und Kinder mit externalisierenden Symptomen erhielten insgesamt mehr Leistungen als Kinder ohne diese Symptome.

## 要旨

本研究は、米国中西部におけるCOVID‐19の流行が幼児のメンタルヘルスおよび精神保健サービス利用に及ぼす影響を調査するものである。COVID‐19が幼児の精神保健サービス利用に及ぼす影響を調査した先行研究では、5歳未満の小児を対象としたものはほとんどなかった。そこで本研究では、COVID‐19の流行と養育者と子どもの関係が、子どもの精神衛生症状にどのような影響を与えたかを検討した。また、COVID‐19と子どもの症状呈示が、養育者のメンタルヘルス治療への関与と出席に及ぼす影響についても調査した。データは、2017年1月から2022年4月にかけて、地域の精神保健組織のアーカイブ記録を用いて0～5歳の子ども(*n* = 486)を対象に収集した。参加者は主に低所得者(81.9％)、白人(81.3％)であった。結果は、養育者と子どもの関係が、COVID‐19パンデミック前とパンデミック中の子どもの精神的健康症状に影響を与えることがわかった。パンデミック開始後、子どもが外在化症状を経験した場合、養育者と子どもの両方がより深く関わっていると評価された。パンデミック開始後、子どもはセラピーセッションに参加する回数が減り、外在化症状のある子どもはそうでない子どもよりも全体としてより多くのサービスを受けていた。

## Resumen

Este estudio investiga el impacto de la pandemia del COVID‐19 en la salud mental de los niños pequeños y su participación en servicios de salud mental en el medio oeste de los Estados Unidos. La investigación previa que se ha enfocado en el impacto del COVID‐19 en la utilización de servicios de salud mental por parte de niños pequeños raramente ha incluido a los niños menores de 5 años. Por tanto, este estudio examinó cómo la pandemia del COVID‐19 y la relación entre el niño y quien le presta el cuidado tuvo impacto sobre los síntomas de salud mental de los niños. También investigó el impacto del COVID‐19 y la presentación de los síntomas del niño sobre la participación y asistencia del cuidador en el tratamiento de salud mental. Se obtuvieron los datos con niños de 0 a 5 años (*n* = 486) desde enero de 2017 a abril de 2022 usando documentos archivados de una organización comunitaria de salud mental. Los participantes eran primordialmente de bajos recursos económicos (81.9%) y blancos (81.3%). A través de los resultados se encontró que la relación entre cuidador y niño tuvo un impacto en los síntomas de salud mental de los niños antes y durante la pandemia del COVID‐19. Tanto a los cuidadores como a los niños se les evaluó como más participativos en la relación después que comenzó la pandemia si el niño experimentaba síntomas externalizantes. Los niños asistieron a menos sesiones de terapia después que comenzó la pandemia, y aquellos con síntomas externalizantes recibieron en general más servicios que aquellos sin esos síntomas.

## 1 COVID‐19 AND CHILDREN'S MENTAL HEALTH

In March 2020, the rapid escalation of the COVID‐19 pandemic abruptly and severely disrupted daily life, leading to prolonged lockdowns, distance learning, mask mandates, and other protective health measures across the entire United States and worldwide. This unprecedented event caused many unknowns, stressors, and increases in mental health concerns for people of all ages throughout the world (World Health Organization, 2022). Children, particularly very young children, are uniquely susceptible to disasters such as COVID‐19 because they have little experience in coping with stressors or asking for help when they are struggling (Silverman & La Greca, 2002). Researchers Silverman and La Greca (2002) note that the impact of disasters may be further exacerbated by the assumptions that children will recover, are resilient, or may not fully grasp the gravity of the situation. While children are resilient in many cases, they may still see long‐lasting psychological effects.

Researchers have since confirmed that children experienced significantly more severe stressors such as family violence (Usher et al., 2020) as well as clinical levels of depression and anxiety during the pandemic compared to estimates before the pandemic (Madigan et al., 2023; Racine et al., 2021). Caregivers who were interviewed in the United Kingdom also reported that children 5 to 18 years who had mental health diagnoses pre‐pandemic experienced worsening symptoms of their already‐present diagnoses (Asbury et al., 2021).

While most previous research has primarily focused on older children, only a few studies have examined the specific effects of the pandemic on younger children's emotional and behavioral health. Early childhood samples collected in Italy showed reported increases in anxiety, depression, inattention, hyperactivity, aggression, and oppositional behavior among children ages birth to six (Cantiani et al., 2021; Di Giorgio et al., 2021). Similarly, Glynn et al., 2021 concluded that preschool children demonstrated both elevated depressive and disruptive behavioral symptoms compared to pre‐pandemic norms. The RAPID Survey Project from the Stanford Center on Early Childhood (2023) also reported that young children experienced significant increases in externalizing and internalizing behaviors at the beginning of the pandemic and that they have remained elevated over the course of the last few years. While these studies confirm the significant effects of the pandemic on the mental health of very young children, the relative number of studies focused on this population is small. This study aims to investigate the nuance that the pandemic had on young children, including how the quality of the caregiver‐child relationship affected young children's mental health and how symptom presentation uniquely affected children's and caregivers’ attendance and engagement in mental health services.

### 1.1 Caregivers’ and children's mental health

When facing significant stressors, caregivers are the key component of young children's coping response (Power, 2004). Contemporary stress models for children depict that when children first encounter a potentially stressful event, they take steps to identify if it is indeed stressful, and then exhibit a behavioral response that may include coping or not coping, and approaching or avoiding the stressful situation (Power, 2004). At every stage in this model, children look to their caregivers for support and understanding. This is especially true with younger children who are more reliant on caregivers for emotional support. These interactions between caregivers and children are bidirectional, such that caregivers may provide a buffer and protection from the stressor. They may do this by limiting the information or by checking in on the child.

**Key Findings**

- Young children were not more likely to demonstrate clinically significant mental health symptoms before or after the pandemic began but were less likely to have clinically significant mental health symptoms if they had more optimal relationships with their caregivers.
- Externalizing symptoms have a unique positive effect on children and their caregivers’ ability to engage in mental health services during the pandemic.
- Children and their caregivers attended 1.6 times more therapy sessions before COVID‐19 began than during COVID‐19.

**Relevance**

This study offers insight into how the COVID‐19 pandemic impacted early childhood mental health and mental health services. The conclusions point to implications for early childhood mental healthcare providers and a greater understanding of barriers to treatment during the COVID‐19 pandemic.

Similarly, research before the pandemic suggested that there was a bidirectional relationship between caregivers’ and children's mental health. Specifically, caregiver distress (Neece et al., 2012), parental depression, (Pardini, 2008; Weissman et al., 1987), and psychological distress (including anxiety, depression, and somatization symptoms; Gewirtz et al., 2009) were found to both influence and be influenced by their children's mental health problems. Parental mental health problems were also associated with disruptions in parenting, which led to negative mental health impacts on children (Smith, 2004). In light of the severe life disruption, family isolation, and stress generated by the COVID‐19 pandemic, this reciprocal influence between caregivers' stress responses and mental health challenges and their children's mental health has been exacerbated (Babore et al., 2023; Patrick et al., 2020; Westrupp et al., 2021). Caregiver increases in depression and anxiety were linked to increases in children's anxiety and depression (Gruhn et al., 2023). Specifically with younger children, an Austrian longitudinal study found increases in preschool‐aged children's mental health symptoms during the pandemic were moderated by maternal mood (Frigerio et al., 2023).

Caregivers also experienced increased parenting stress during the pandemic, in part due to increasing children's emotional and behavioral distress (Babore et al., 2023; Sahithya et al., 2020; Westrupp et al., 2021). This increase in parenting stress was associated with small to moderate increases in aggressive discipline behaviors such as spanking or slapping (Sahithya et al., 2020). Higher levels of aggressive and negative parenting behaviors have been previously associated with higher levels of distress and mental health problems in children (Berg‐Nielsen et al., 2002; Hosokawa & Katsura, 2018; Stormshak et al., 2000).

### 1.2 Caregiver‐child relationship and children's mental health

Beyond investigating the bidirectional relationship between children's and caregivers’ mental health, it is critical to examine how the caregiver‐child relationship has been affected by the pandemic. In turn, it is important to emphasize how the caregiver‐child relationship could act as a risk or protective factor for children and their mental health against the pandemic depending on its quality. Some disaster‐related research previous to the pandemic suggested that the quality of caregiver‐child relationships or strong family relationships in general moderated the effect that the disaster had on children's mental health symptoms, in that those with higher quality relationships with their caregiver or stronger family ties experienced less distress with small to moderate effect sizes (Osofsky et al., 2015; Qouta et al., 2008; Wickrama & Kaspar, 2007).

A few studies appear to have investigated the effects that the COVID‐19 pandemic had on the caregiver‐child relationship and children's mental health, and the results were somewhat mixed. Some evidence suggests that the pandemic resulted in positive changes to the caregiver‐child relationship with children under the age of 15 (Sahithya et al., 2020). Others reported lower positive family expressiveness in general (Westrupp et al., 2021). Russell & colleagues (2020) investigated the parent‐child relationship in children under 18 years old and found that parent‐child relationship conflict was correlated with higher levels of generalized anxiety, depression, caregiver burden, and parental perceived child stress, whereas child‐parent closeness was significantly negatively associated with those variables. This was consistent with the finding that increases in family conflict during the pandemic predicted higher internalizing and externalizing symptoms in 4‐year‐old children (Fosco et al., 2022). However, there is still little data about how the caregiver‐child relationship affected young children's mental health during the pandemic.

### 1.3 Engagement and attendance in mental health services during COVID‐19

In summary, children were in greater need of mental health services during the COVID‐19 pandemic. However, the pandemic and public safety measures affected how mental health services were delivered, which in turn affected attendance and engagement. Before the pandemic, previous research suggested that in general, 28% to 75% of children prematurely drop out of mental health services due to a variety of barriers and concerns (de Haan et al., 2013). Specifically, children with higher externalizing symptoms were more likely to prematurely drop out of services (de Haan et al., 2013; Eslinger et al., 2014; Grassetti et al., 2018; Wamser‐Nanney & Steinzor, 2016). Overall, this data suggested that children were not receiving the help that they needed even before the pandemic affected mental health service delivery.

Caregiver engagement in mental health services is critical for children's initial access to services, effective continuation to termination, and successful treatment outcomes (de Greef et al., 2017; Haine‐Schlagel et al., 2018, 2017; Kirsch et al., 2018; Retamal‐Walter et al., 2023). In pre‐pandemic studies, caregivers were more likely to engage in services if their child was experiencing externalizing behaviors (Stadnick et al., 2016; Wright et al., 2019), or if the caregivers were experiencing greater parenting stress (Lai et al., 2019), which is often associated with greater externalizing behaviors (Mackler et al., 2015). Consequently, caregivers are an important part of children's therapy attendance and outcomes and likely determine whether children get the help they need.

During the early part of the pandemic, mental health services were initially suspended, leaving families without care for a few weeks. Later, services transitioned to telehealth, in which children and caregivers video or audio chatted with their mental health providers. Telehealth for children and adolescents is comparable to in‐person services in many ways (Gloff et al., 2015) and is effective across diagnoses and diverse populations (Nelson et al., 2016). Telehealth may also increase accessibility for some families who live in rural communities, and decrease the cost of travel, time spent traveling to sessions, and stress associated with traveling to services (Nelson et al., 2016). Early qualitative data suggested an increase in regular attendance by youth in therapy during the COVID‐19 pandemic (Schriger et al., 2022; Walters, 2021) as well as higher levels of caregiver engagement (Schriger et al., 2022).

In surveys and interviews with 50 caregivers with children in outpatient telehealth therapy, data revealed that all 50 were satisfied with the services they were receiving and 42% even reported that they prefer telehealth (Moorman, 2021). Clinicians in this same study reported that they felt that adolescents opened up more about their difficulties, but that engaging younger children was considerably harder in the virtual format. Attendance rates for outpatient programs in this study increased from 74% to 89% through the pandemic as well.

However, engagement over telehealth can be difficult. Attention and behavioral difficulties make focusing online particularly challenging for young children (Racine et al., 2020; Walters, 2021). Engaging younger children on an online platform is also difficult in general (Burns et al., 2023; Schriger et al., 2022). Other barriers to telehealth for children and adolescents include a lack of a safe and confidential environment at home, devices and broadband networks, and caregiver knowledge of the telehealth platforms (Palinkas et al., 2021). If children and their caregivers are unable to access the internet, they will be unable to engage in therapy sessions. Knowing that telehealth was shown to help reduce barriers and potentially increase attendance but was also difficult with certain groups of children warrants further investigation into how children and their families were engaging and attending services during the pandemic, especially in the early childhood population.

### 1.4 Current study and hypotheses

The current study investigated the impact that COVID‐19 had on a sample of children in an in‐home early childhood mental health program (0–5 years old) in the Midwest. The study compared a group of young children who received services before the COVID‐19 pandemic to a separate independent group that received services during the COVID‐19 pandemic in a between‐subjects design. Moreover, this study aimed to understand how the pandemic and social distancing measures impacted engagement and attendance in early childhood mental health services.

Based on the pre‐pandemic research and early reports from the pandemic, three different hypotheses and models were created. Hypothesis 1 predicted that children receiving services before the pandemic who also had higher‐quality relationships with their caregivers would be less likely to exhibit clinically severe symptoms than children receiving services after the pandemic began. Hypothesis 2 stated that young children receiving services during the pandemic who also exhibited clinically significant externalizing symptoms were less engaged in mental health services than children receiving services before the pandemic without externalizing symptoms. Based on the prediction that children were less engaged, it was further hypothesized that caregivers of children experiencing clinically significant externalizing symptoms were more engaged in sessions during the COVID‐19 pandemic. Finally, Hypothesis 3 stated that children and their caregivers attended fewer sessions if the child was demonstrating externalizing symptoms than children without externalizing symptoms because previous research suggested that externalizing behaviors predicted treatment dropout.

## 2 METHOD

### 2.1 Participants

The participants in this study included 486 children who started services in an early childhood community mental health program from January 2017 through April 2022. Participants consented to future research studies using their de‐identified data when they signed an informed consent document at the beginning of treatment. Archival data were collected by the participating clinic, redacted to remove personal identifying information, and sent to the researchers via an encrypted email. This study was approved by the university's Institutional Review Board. See Table 1 for demographic information of the participants.

**TABLE 1:** Demographic characteristics.

Variable	*n*	%	*M*	SD
Age			3.62	1.06
Insurance				
MA/PMAP/Grants	281	81.9		
Private	62	18.1		
Ethnicity				
Not of Hispanic or Latina/o/x/e	323	94.2		
Hispanic or Latina/o/x/e	13	3.7		
Unknown	7	2.1		
Race				
American Indian/ Alaskan Native	27	7.9		
Asian American	1	.3		
Black/African American	21	6.1		
Multi‐Racial	8	2.3		
Native Hawaiian/ Other Pacific Islander	1	.3		
White	279	81.3		
Unknown	2	.6		
Sex Assigned at Birth				
Male	195	56.9		
Female	148	43.1		

### 2.2 Measures

Basic information regarding each participant's age, sex assigned at birth, diagnosis, race/ethnicity, and treatment dates were collected for demographic and descriptive statistical purposes. The categories of the demographic data were based on the data file. Socioeconomic status was identified through the proxy variable of insurance type and was sorted into two groups. The two groups were defined as a lower‐income group that received medical assistance or grants to cover their services and a higher‐income group with private insurance coverage.

### 2.3 Caregiver‐child relationship

The Early Childhood Service Intensity Instrument (ECSII; American Academy of Child and Adolescent Psychiatry, 2009) was administered at or near the start of treatment to assess the caregiver‐child relationship. The ECSII is a 48‐question assessment that is divided into six domains, including Degree of Safety, Child‐Caregiver Relationship, Caregiving Environment, Functional/Developmental Status, Impact of the Child's Medical, Developmental, or Emotional/Behavioral Problems, and Service Profile. For all of the subscales, higher scores reflect higher levels of dysfunction and distress in a child's life and therefore a greater need for services. There is one overall service intensity score set up in a Likert scale format from 0 (*basic health care*) to 5 (*full support*). The child‐caregiver relationship domain is specifically measured on a scale of 1 (*optimal;* e.g., “the relationship is functioning well and is consistently satisfying to both caregiver and child”) to 5 (*severe impairment;* e.g., “the relationship is severely disturbed and distressing to the caregiver and child such that the child is in imminent danger of physical harm”). This measure has sufficient interrater reliability, concurrent validity, and criterion validity (American Academy of Child and Adolescent Psychiatry, 2006). Individual items were not recorded in the participants’ medical records and could therefore not be used to determine internal reliability within the study sample.

### 2.4 Engagement and attendance in services

The ECSII (American Academy of Child and Adolescent Psychiatry, 2009) was also used to identify clinicians’ perceptions of each child's as well as their caregiver's engagement with services, which fall under the service profile domain. The Service Profile domain is further divided into four categories of questions, including Caregiver(s) Involvement in Services, Child's Involvement in Services, Service Fit, and Effectiveness of Services. Questions within the Caregiver(s) Involvement in Services and the Child's Involvement in Services were used to assess engagement. These two groups of questions are measured on a scale of 1 (*optimal;* e.g., “child is fully engaged during all interactions with provider(s) in an age‐appropriate manner.”) to 5 (*none;* e.g., “caregiver(s) and providers have complete disagreement about the child and family's strengths and needs regarding the child's service plan”).

The number of therapy sessions was also utilized to assess treatment engagement. Because the ECSII engagement section is limited to a five‐point scale with only moderate interrater reliability, measuring engagement through attendance allowed for a greater understanding of how the pandemic affected the number of sessions that participants were receiving or engaging in and how this measure may relate to other variables. Attendance is an objective measure of overall treatment adherence and engagement. This data was only collected and analyzed with children who terminated therapy during data collection, either because they chose to terminate or because the termination was mutual between the therapist and the family.

### 2.5 Internalizing and externalizing behaviors

The Childhood Behavior Checklist for ages 1.5‐5 (CBCL/1.5‐5; Achenbach & Rescorla, 2000) was used to assess symptom severity at the start of treatment. The CBCL/1.5‐5 is completed by caregivers at the start of treatment to aid in diagnosis and understanding of the child's current symptom presentation. Caregivers rate the 99 behavior questions on a scale of 0 (*Not true as far as you know*) to 2 (*Very true or often true*). For this study, data for this variable was reported by the agency as to whether or not the child's symptoms met clinical significance on the DSM‐Oriented Scale, which includes five categories: Affective Problems, Anxiety Problems, Pervasive Developmental Problems, Attention Deficit/Hyperactivity Problems, and Oppositional Defiant Problems. This scale was chosen for its fidelity to diagnostic codes. The behavior subscores are deemed clinically significant if above the 98th percentile (T‐score of approximately 70). Further, cases were re‐coded to reflect externalizing, internalizing, or other types of symptom presentations. Externalizing concerns was the focus of this re‐code. Children who were indicated to have attention deficit hyperactivity disorder or oppositional defiant problems were considered to have externalizing presentations. All other symptom presentations were put into the not externalizing presentation category. This included affective problems, anxiety problems, pervasive developmental problems, and No Significant DSM‐Oriented Scale. This measure has excellent internal consistency, test‐retest reliability, and good discriminant and predictive validity (Achenbach & Rescorla, 2000). Individual item ratings were not reported in the medical records used as part of this archival study, therefore internal reliability estimates could not be analyzed for this data. Validity of the CBCL1.5/5 reported scores were assessed by comparing clinically significant status of the sample to their ECSII caregiver‐child relationship scale. Results indicate a significant association between these measures, *χ* ^2^ (3) = 19.55, *p* < .001, where children rated as having clinically significant symptoms on CBCL1.5/5 were more likely to have impairment noted within the caregiver‐child relationship.

### 2.6 Data analyses

#### 2.6.1 Defining variables

Table 2 displays the frequencies of each level of the categorical variables for this study. Whether the case was considered to be in the before COVID‐19 or during COVID‐19 group depended on the hypothesis outcome variable. The distinction between the two different COVID‐19 definitions was made because some cases would be placed in the before‐COVID group based on the administration date of the intake measures (ECSII and CBCL1.5/5), but may have received the majority of their therapy services during COVID‐19. The first two hypotheses measured the moment in which the measures were completed, whereas the last hypothesis measured the attendance over time. For the first two hypotheses, COVID‐19 status was determined by the date of the ECSII and CBCL1.5/5. For the last hypothesis, COVID‐19 status was determined by the amount of time the client was in services before or after March 15, 2020. If the majority of the time in services was before March 15, 2020, they were placed into the before‐COVID group. If the majority of the time was after March 15, 2020, they were placed into the during‐COVID group.

**TABLE 2:** Frequencies and descriptive statistics for key outcomes.

Variable	*n*	%	*M*	SD
Therapy Sessions			6.48	9.88
All Services			29.25	49.97
COVID Status Based on ECSII/CBCL				
Before	315	64.8		
After	171	35.2		
COVID Status Based on Attendance				
Before	287	59.1		
After	199	40.9		
ECSII Caregiver‐Child Relationship				
Optimal	15	3.1		
Adequate	171	35.2		
Mild Impairment	262	53.9		
Moderate Impairment	38	7.8		
Severe Impairment	0	0		
ECSII Child Engagement				
Optimal	17	3.5		
Adequate	328	67.5		
Limited	115	23.7		
Minimal	13	2.7		
None	13	2.7		
ECSII Caregiver Engagement				
Optimal	52	10.7		
Adequate	341	70.2		
Limited	72	14.8		
Minimal	10	2.1		
None	11	2.3		
CBCL Symptom Presentations^a^				
Externalizing	157	32.3		
Internalizing	161	33.1		
Both Internalizing and Externalizing	21	6.1		
Other	93	19.1		
Externalizing and Other	11	3.2		
Internalizing and Other	23	6.7		
None	221	46.4		
All Three Symptom Types	35	10.2		

^a^ Percentage of cases that fell into the clinical range of the CBCL1.5/5.

**TABLE 3:** Results of the Binary Logistic Regression for Clinically Significant Mental Health Symptoms.

						95% C.I. for Odds Ratio	
Model	*B*	SE	Wald	*p*	Odds Ratio	Lower	Upper
1							
COVID‐19 Status^a^	.05	.20	.06	.81	1.05	.70	1.58
2							
COVID‐19 Status	.27	.22	1.48	.22	1.31	.85	2.01
C‐C Relationship			20.03	.001			
C‐C Relationship (1)	1.00	.68	2.17	.14	2.73	.72	10.32
C‐C Relationship (2)	1.80	.67	7.11	.008	6.03	1.61	22.61
C‐C Relationship (3)	2.01	.76	7.06	.008	7.47	1.70	32.96
3							
COVID‐19 Status	1.54	1.41	1.20	.27	4.67	.30	73.38
CC Relationship			17.22	<.001			
C‐C Relationship (1)	1.55	1.09	2.01	.16	4.71	.55	40.12
C‐C Relationship (2)	2.50	1.08	5.35	.02	12.17	1.47	101.11
C‐C Relationship (3)	2.59	1.14	5.17	.023	13.30	1.43	123.79
COVID‐19 by Caregiver Child Relationship Interaction			2.08	.56			
COVID‐19 by Caregiver Child Relationship Interaction (1)	−1.09	1.44	.57	.45	.34	.02	5.71
COVID‐19 by Caregiver Child Relationship Interaction (2)	−1.55	1.44	1.16	.28	.21	.013	3.58
COVID‐19 by Caregiver Child Relationship Interaction (3)	−.796	1.84	.19	.67	.45	.01	16.56

^a^ COVID status was determined by whether the ECSII and CBCL1.5/5 were administered before or after March 15, 2020.

In addition, for the third hypothesis, the dataset revealed many different types of services each child received during their time in mental health services. To understand how many sessions the children and their caregivers engaged in, it was decided that two different analyses were to be run to understand the nuances of this data. The first analysis was run using all services that children and their families received (e.g., case consultation, coordination of services, skills sessions, therapy). The hypothesis remained the same—children would be attending and engaging in more services after COVID‐19 began than before. A second analysis was run using only therapy sessions as the outcome variable, which excluded all other types of services. Therapy sessions may require greater engagement since the family has to either welcome the therapist into their home or attend online via telehealth. Other service types, such as consultation, do not require attendance or engagement by the children and their families. The types of services and therapy session types are listed in Appendix A.

### 2.7 Missing data analyses

Missing data analyses revealed approximately 36% of the cases had missing data, including nearly 14% of all values across all variables. Further analysis indicated that children with missing data had significantly fewer sessions, *t* (422) = 4.97, *p* < .001. They were also more likely to be in the during‐COVID group based on the ECSII and CBCL1.5/5, *χ*2 (1) = 12.02, *p* < .001. Finally, cases that were missing data were more likely to fall into the limited/minimal category for scores on the caregiver engagement scale of the ECSII, *χ*2 (1) = 4.63, *p* = .03. As this indicated the data was not missing at random (NMAR), a multiple imputation method was used to estimate missing values, with the pooled data being utilized for all study analyses (Abonazel & Ibrahim, 2018).

## 3 RESULTS

### 3.1 Hypothesis 1: Clinically significant mental health symptoms

For the first and second hypotheses, 49 cases where the ECSII and CBCL1.5/5 were completed more than 2 months apart were removed. This is because the analyses attempted to capture a single moment when assessment measures were completed toward the start of therapy. Then, a binary logistic regression model (*n* = 437) was run to determine the effect of COVID‐19 and the caregiver‐child relationship on clinically significant mental health symptoms. Results of the omnibus test revealed that COVID‐19 status was not a significant predictor of clinically significant mental health symptoms, χ^2^ (1) = .06, *p* = .81. However, the caregiver‐child relationship was found to be a significant predictor, χ^2^ (3) = 21.52, *p* < .001. This model with the single predictor of the caregiver‐child relationship predicted 61% of the variance beyond when the model included only the intercept. The interaction between COVID‐19 status and the caregiver‐child relationship did not significantly improve the model, χ2 (3) = 2.14, *p* = .54, than the caregiver‐child relationship alone to predict clinically significant mental health symptoms. Planned comparisons indicated that children with optimal relationships with their caregivers were significantly more likely to have non‐significant clinical symptoms compared to those who had mild to moderately impaired relationships across both COVID‐19 conditions (See Table 3).

### 3.2 Hypothesis 2: Engagement

#### 3.2.1 Caregiver engagement

An ordinal logistic regression model (*n *= 437) was run to determine the effect of COVID‐19 and externalizing symptoms on caregiver engagement (formatted as an ordinal dependent variable) in mental health services. Significant model fit indices suggest that the predictors were significantly better than the null model at predicting caregiver engagement, χ^2^ (3) = 15.32, *p* = .002. Omnibus tests indicate that COVID‐19 status was a significant predictor, with a parameter estimate of 1.18, Wald statistic = 9.19, *p* = .002. Externalizing symptoms did not significantly predict caregiver engagement, with a parameter estimate of .433, Wald statistic = 1.20, *p* = .27. The interaction between COVID‐19 and externalizing symptoms was significant, with a parameter estimate of −1.44, Wald statistic = 8.83, *p* = .003. Caregivers of young children with externalizing behaviors were more likely to be engaged in treatment during COVID‐19 compared to caregivers of similar children prior to COVID‐19 (See Table  4).

#### 3.2.2 Child engagement

A second ordinal logistic regression model (*n *= 437) was tested to determine the effect of COVID‐19 and externalizing symptoms on children's engagement in mental health services. Significant model fit indices suggest that the predictors were significantly better than the null at predicting child engagement χ^2^ (3) = 14.51, *p* = .002. Omnibus tests show that COVID‐19 status was not a significant predictor of children's engagement, with a parameter estimate of .644, Wald statistic = 3.08, *p* = .085. Externalizing symptoms were also not a significant predictor of children's engagement, with a parameter estimate of −.029, Wald statistic = .006, *p* = .94. The interaction between COVID‐19 and externalizing symptoms was significant, with a parameter estimate of −.975, Wald statistic = 4.44, *p* = .035. (See Table 5). Similar to caregiver engagement, young children were rated by the therapist as being more engaged in therapy if they had significant externalizing behaviors during COVID‐19 compared to similar children prior to COVID‐19.

**TABLE 4:** Results of the ordinal logistic regression for caregiver engagement.

					95% C.I. for Parameter Estimate	
Model	*B*	SE	Wald	*p*	Lower	Upper
1						
COVID‐19 Status^a^	1.18	.39	9.19	.002	.42	1.94
2						
Externalizing Symptoms	.43	.40	1.20	.27	−.34	1.20
3						
COVID‐19 by Externalizing Symptoms Interaction	−1.44	.49	8.83	.003	−2.39	−4.90

^a^ COVID status was determined by whether the ECSII and CBCL1.5/5 were administered before or after March 15, 2020.

### 3.3 Hypothesis 3: Attendance

Before analyzing data distribution qualities, the 49 cases that were excluded from the first two hypotheses were added back in. This is because the third hypothesis was not attempting to capture a moment in time and the ECSII was not used in this analysis. Then, 22 cases were deleted because the children and caregivers were still actively receiving services. Therefore, these children had no discharge date as of the cut‐off date of the records acquisition. Since attendance was utilized as a proxy measure for therapy engagement, a direct comparison of youth and families who had formally completed therapy was most appropriate. The data for attendance demonstrated a significant skew for both total therapy sessions and total services. A square root transformation was used for both variables before the analyses were performed to improve the distribution of the data to fit assumptions of normality.

#### 3.3.1 Total therapy sessions

A 2 × 2 between‐subjects factorial ANOVA (*n *= 464) was run to determine the effect of COVID‐19 and externalizing symptoms on the total number of therapy sessions families received in the early childhood mental health program. Levene's test revealed that the homogeneity of variance assumption was not met (*p* < .001), therefore Welch's test was conducted, in addition to effect sizes, which are more robust to violations of the assumption of homogeneity of variances. The results revealed a significant main effect for COVID‐19, Welch's *F* (1, 431) = 6.30, *p* = .012, *η_p_* ^2^ = .012. An examination of the means and standard deviations revealed that the before COVID‐19 group had significantly more therapy sessions (*M* = 7.56, *SD* = 10.89) than those in the during‐COVID‐19 group (*M* = 4.73, SD = 7.70). The main effect for externalizing symptoms was not statistically significant, Welch's *F* (1, 317) = .33, *p* = .57, *η_p_* ^2^ < .01. Lastly, the interaction effect between COVID‐19 and externalizing symptoms was also not significant, *η_p_* ^2^ < .01 (See Table 6).

**TABLE 5:** Results of the ordinal logistic regression for child engagement.

					95% C.I. for Parameter Estimate	
Model	*B*	SE	Wald	*p*	Lower	Upper
1						
COVID‐19 Status^a^	.64	.37	3.08	.085	−.08	1.36
2						
Externalizing Symptoms	−.03	.385	.006	.94	−.78	.72
3						
COVID‐19 by Externalizing Symptoms Interaction	−.98	.46	4.44	.04	−1.88	−.07

^a^ COVID status was determined by whether the ECSII and CBCL1.5/5 were administered before or after March 15, 2020.

#### 3.3.2 Total services

A between‐subjects factorial ANOVA (*n *= 464) was run to determine the effect of COVID‐19 and externalizing symptoms on the total number of services families received in the early childhood mental health program. Levene's test revealed that the homogeneity of variance assumption was again not met (*p* < .001), therefore Welch's *F* statistic and effect sizes will be reported. Omnibus tests did not reveal a significant main effect for COVID‐19, Welch's *F* (1, 413) = 1.22, *p* = .27, *η_p_* ^2^ = .03. The main effect for externalizing symptoms was significant, Welch's *F* (1, 282) = 4.07, *p* = .05, *η_p_* ^2^ = .08. Children exhibiting externalizing behavioral symptoms (*N* = 154) received more overall services (*M* = 34.96, *SD* = 56.59) compared to children without externalizing symptoms (*N *= 310, *M* = 26.42, *SD* = 46.17) across all COVID‐19 conditions. Lastly, the interaction effect between COVID‐19 and externalizing symptoms was also not significant, *η_p_* ^2^ < .01. See Table 7 for additional information.

**TABLE 6:** ANOVA results for total number of therapy sessions.

Predictor	Sum of Squares^b^	df	*F* ^c^	*p*	*ηp2*
Intercept	1137.34	1			.42
COVID‐19 Status^a^	19.85	1	6.30	.012	.01
Externalizing Symptoms	.66	1	.33	.57	<.001
COVID‐19 by Externalizing Symptoms Interaction	.85	1			<.001

^a^ COVID status was determined by the amount of time the client was in mental health services before or after March 15, 2020.

^b^ Sum of squares reflects the square root skew transformation.

^c^ Welch's *F* statistic.

## 4 DISCUSSION

This study had two intentions. The first was to identify the impact of COVID‐19 and the caregiver‐child relationship on mental health symptoms in young children. This study did not confirm the hypothesis or align with previous research that children were more likely to have clinically significant mental health symptoms after the COVID‐19 pandemic began (Madigan et al., 2023; Racine et al., 2021). A potential explanation for this finding is that the data was collected too soon after the pandemic began. Younger children may display more mental health difficulties in their elementary years when increased social demands and coping strategies are necessary (Davies & Troy, 2020), which may have not developed while social distancing at home in early childhood. This may lead to increased mental health symptoms in the future (Almeida et al., 2021). Another possible explanation is that children with more significant mental health concerns were less likely to be able to attend or engage in services in general (Bringewatt & Gershoff, 2010), which may have contributed to the similarities of the two groups in terms of clinically significant mental health concerns. More specifically, it is known that children in this data set with missing data were more likely to be in the during‐COVID group. Despite utilizing multiple imputation to address the impact of missing data, this could have contributed to the lack of nuance between the groups as cases with imputed data were more likely to be in the during‐COVID group.

**TABLE 7:** ANOVA results for total number of services.

Predictor	Sum of Squares^b^	df	*F* ^c^	*p*	*ηp2*
Intercept	6458.34	1			.52
COVID‐19 Status^a^	15.29	1	1.22	.27	.003
Externalizing Symptoms	51.29	1	4.07	.05	.008
COVID‐19 by Externalizing Symptoms Interaction	.44	1			<.001

^a^ COVID status was determined by the amount of time the client was in mental health services before or after March 15, 2020.

^b^ Sum of squares reflects the log skew transformation.

^c^ Welch's *F* statistic.

The study confirmed the hypothesis that the caregiver‐child relationship affected early childhood mental health symptoms. Children with more optimal relationships with their caregiver were less likely to demonstrate clinically significant mental health symptoms, and children with less optimal relationships were more likely to demonstrate clinically significant mental health symptoms. This finding agrees with previous studies that have deemed the caregiver‐child relationship as a protective factor for children, especially regarding their ability to cope during potentially stressful events (Osofsky et al., 2015; Power, 2004; Proctor et al., 2007). Taken together, children with optimal caregiver‐child relationships may have continued to experience those relationships as protective during the pandemic, while those with less optimal relationships continued to struggle.

The second intention was to identify the impact of COVID‐19 and externalizing symptoms on engagement and attendance in an early childhood mental health program. This study found that overall caregivers were rated as less engaged in services during COVID regardless of their child's symptom presentation when these two variables were isolated. However, when considering the interaction between these two concepts, the study found that both caregivers and children were rated as more engaged after the pandemic began if the child had externalizing symptoms. For caregivers, these findings are somewhat consistent with previous research. Reports pre‐pandemic suggested that caregivers were more engaged when their children were experiencing externalizing symptoms (Stadnick et al., 2016; Wright et al., 2019), potentially due to increased parenting stress (Lai et al., 2019) and that they were more engaged during the pandemic in general (Schriger et al., 2022). Alternatively, caregivers were rated as more engaged after the pandemic began because their children were also rated as more engaged after the pandemic began. Together, they were engaging more optimally. The pandemic may have also promoted engagement for caregivers of children with externalizing behaviors as they may have had to be more attentive to how their child was engaging over telehealth compared to in‐person services (Wright et al., 2019).

The present study found COVID‐19 by itself did not have significant impacts on engagement for children. Previous research suggested that young children with externalizing symptoms have a difficult time engaging in services, especially over telehealth (Racine et al., 2020; Schriger et al., 2022; Walters, 2021). In addition, while there is some evidence to support the telehealth delivery of several empirically supported treatments utilized by practitioners in this study, (e.g., Triple P, Attachment and Biobehavioral Catch‐Up, TF‐CBT, Family Therapy), samples of these studies do not consistently include children in the birth to five age range, which may contribute to the variability in these findings (Labella et al., 2023; Maguire‐Jack et al., 2022; McLean et al., 2021; Reese et al., 2015; Stewart et al., 2020). In this study, children were rated as more engaged during the pandemic, especially if they had externalizing symptoms. As already noted, this finding could be related to caregivers also being rated as more engaged during the pandemic. In conclusion, the present study suggested that caregivers engaged in services when their child with externalizing problems was also more optimally engaging in services during the pandemic. In other words, a child's externalizing behavior uniquely and positively affected families’ abilities to engage in services during COVID‐19.

Attendance was also investigated to gain more insights into families’ engagement with mental health services before and during the COVID‐19 pandemic. The present study revealed that children received a greater number of therapy sessions before the COVID‐19 pandemic began than during the pandemic. This is contrary to the hypothesis and previous qualitative data that children were attending more sessions when the COVID‐19 pandemic began (Schriger et al., 2022; Walters, 2021). This finding may suggest that families had difficulties attending services through telehealth or may have scheduled less sessions due to barriers (Lyzwinski et al., 2024). Families may have made this decision thinking that their young child would have difficulties attending online.

Results also indicated that children received a similar number of overall services before and during the COVID‐19 pandemic, which is in contrast to previous research findings that mental health services decreased significantly during the pandemic (Stewart et al., 2022). This suggests that children and their families may have asked for similar numbers of services regardless of the time point. Another possible explanation is that mental health practitioners were identifying and supporting the needs of families and documenting those activities at the same rate both before and during COVID‐19 following effective policy changes that removed barriers to accessing telehealth services (Palinkas et al., 2021). However, families received more services if the child was experiencing externalizing symptoms across both time periods. Mental health providers may have been more responsive to the needs of families with a child with externalizing symptoms across both time periods by providing a wider range of supportive services. This may be due to the likelihood of increased parenting stress (Lai et al., 2019).

In summary, children and their caregivers were rated as more engaged in services if the child was experiencing externalizing symptoms during the pandemic. Overall, though, families received fewer therapy sessions during the pandemic. These families of children with externalizing symptoms were also receiving more overall support in terms of the number of services documented in both time periods. This suggests that externalizing symptoms uniquely impacts the number of overall services given to families, but not necessarily the number of therapy sessions. A possible explanation is that due to the shortage of therapists in many areas (Health Resource & Service Administration, 2024), more services may have been offered by non‐licensed professionals. These non‐licensed professionals in this study may not have provided therapy, but were still offering direct support to children and their families, leading to more documented services.

### 4.1 Strengths and limitations

A strength of this study is that it adds to the growing literature on the pandemic's unique impact on the youngest children. This study also investigates children specifically seeking services at an outpatient mental health clinic and therefore includes a clinical sample that may be uniquely vulnerable to the impacts of the pandemic. In addition, this study investigates the impact of symptom presentation on attendance and engagement in services, which can provide important information to clinicians seeking to engage young families in services.

This study does include some limitations. It is possible that the study was underpowered based on priori power analyses (Bujang et al., 2018). There were also high numbers of participants missing a significant number of relevant data points as well as significant missing data points on many of the variables. A multiple imputation method was used to decrease the effect of the missing data, but the data was not missing at random. Therefore, there may continue to be bias in the data.

In addition, the sample was primarily White, non‐Hispanic, and low‐income. This sample represents the midwestern area that the partnering organization serves. However, generalizability to the greater population beyond this geographical area may be questioned.

A final consideration for this study is that the data was collected until April 2022. Mental health practitioners began seeing clients back in person at differing times, and this may have oscillated between telehealth and in‐person over time due to COVID‐19 exposures and sickness. This may have affected engagement and attendance in services. The data did not provide specific dates of services of when telehealth or in‐person services occurred. Therefore, the patterns and impact of telehealth are unknown.

### 4.2 Clinical implications

The findings from this study point to important implications for early childhood mental health service providers. This study continues to support the idea that the caregiver‐child relationship is a key factor in young children's mental health. The data also suggests that many of those seeking treatment have mild impairments in these relationships. This advocates for mental health practitioners to be attuned to the relationship between caregivers and their children and may see greater benefits from choosing attachment‐based interventions to promote positive caregiver‐child relationships.

Further, the results find that children and their families received similar numbers of overall services during and before the pandemic. This could point to the feasibility of remote services and other adaptations that were made during the pandemic in allowing young children and their families to continue to be connected to mental health services despite barriers that the COVID‐19 pandemic social distancing restrictions created. Telehealth therefore should continue to be a potential option to engage families in mental health services. In addition, mental health providers may benefit from being aware that more services are provided to families of children with externalizing symptoms, either because more services are requested or seen as beneficial for these families.

### 4.3 Future directions

Future research could aim to replicate and extend this study. This could include other measures of mental health symptoms and mental health service engagement, including multi‐informant data. This study included only one data source for each construct, so future studies may benefit from gaining perspectives of engagement from caregivers themselves as well as mental health providers’ perspectives on a child's mental health symptomatology. Further, it is unknown why families may be more or less engaged. This may be an important consideration for future research as well.

Future research would also benefit from including trauma as another covariate. The CBCL1.5/5 does not capture whether the externalizing symptoms may have been related to trauma or another mental health concern. A trauma‐specific scale may create greater nuance in understanding how externalizing symptoms as a result of trauma may have led to greater or lesser engagement or attendance. It may also provide additional understanding of the need for trauma‐specific services at this time. Other variables that could be investigated are electronic device access, parenting stress, and caregivers’ mental health distress. It may also be beneficial to investigate the impact of variables such as race, ethnicity, socioeconomic status, and gender on these outcomes as well. Future research could continue to study this younger population longitudinally as well. This may confirm or disconfirm the hypothesis there was not a difference in mental health symptoms between the two groups because COVID‐19 and social distancing will have a latent effect on those who experienced the pandemic in early childhood.

## CONFLICT OF INTEREST STATEMENT

The authors declare no conflicts of interest.

## ETHICAL APPROVAL

This study was approved by the University of Minnesota Institutional Review Board.

## Data Availability

The data for this manuscript are stored on a secure server and available from the corresponding author upon reasonable request.

## APPENDIX A

### Types of services

#### Types of therapy

Attachment and Biobehavioral Catchup Family Therapy with Patient
Family Therapy with Patient
Family Therapy without Patient
Individual Therapy
Telehealth Supervised Attachment and Biobehavioral Catchup—Family With Patient
Telehealth Supervised Trauma‐Focus Cognitive Behavioral Therapy—Family Without Patient
Telehealth Trauma Informed Child‐Parent Psychotherapy—Family with Patient
Telehealth Trauma Informed Child‐Parent Psychotherapy—Family Without Patient
Telehealth‐Family Therapy with Patient
Telehealth‐Family Therapy Without Patient
Telehealth‐Individual Therapy
Parent‐Child Interaction Therapy—Family with Patient
Parent‐Child Interaction Therapy—Family without Patient
Parent‐Child Interaction Therapy—Family without Patient
Parent‐Child Interaction Therapy—Individual
Supervised Attachment and Biobehavioral Catchup Family Therapy with Patient
Supervised Crisis Therapy
Supervised Family Therapy with Patient
Supervised Family Therapy without Patient
Supervised Family Without Patient—Telehealth
Supervised Individual Therapy
Supervised Trauma‐Focus Cognitive Behavioral Therapy—Family with Patient
Supervised Trauma‐Focus Cognitive Behavioral Therapy—Individual
Supervised Trauma Informed Child‐Parent Psychotherapy—Family with Patient
Supervised Trauma Informed Child‐Parent Psychotherapy—Family without Patient
Supervised‐Individual Therapy Telehealth
Trauma‐Focus Cognitive Behavioral Therapy—Family with Patient
Trauma‐Focus Cognitive Behavioral Therapy—Family without Patient
Trauma‐Focus Cognitive Behavioral Therapy—Individual
Trauma Informed Child‐Parent Psychotherapy—Family with Patient
Trauma Informed Child‐Parent Psychotherapy—Family without Patient
Trauma Informed Child‐Parent Psychotherapy—Individual
Telehealth Attachment and Biobehavioral Catchup Family Therapy With Patient
Supervised—Group Therapy
Supervised Triple P Group
Triple P Group

#### Other services indicated in chart

Administration
Assessment
Care Coordination by Phone or Electronic with Client
Care Coordination by Phone or Electronic with Provider
Care Coordination Face to Face with Client
Case Coordination
Clinical Care Consultation, Face to Face
Clinical Care Consultation, Phone
Comprehensive Evaluation
Comprehensive Integrated Treatment Plan Review/Update
Contact Attempt
Coordination of Care
Coordination of Care—Other Provider
Coordination of Care—Parent
Coordination of Care—School Staff
Court Consultation
Court Testimony—Late Cancel
Crisis Assistance
Children's Therapeutic Services and Supports Assessments and Standardized Measures
Children's Therapeutic Services and Supports Crisis Assistance
Children's Therapeutic Services and Supports Group Skills
Children's Therapeutic Services and Supports Treatment Plan Development and Review
Diagnostic Assessment Extended
Diagnostic Assessment Extended Non billable
Diagnostic Assessment Standard
Evaluation and Management Established Patient
Evaluation and Management New Patient
Employee Assistance Program
Face to Face with Client Non Billable
Face to Face with Provider
Family Skills Training
Family Skills Training Non billable
Functional Assessment
Group Skills
Group Skills Non Billable
Individual Community Support Plan
Indirect Contact
Indirect Note
Individual Skills Training
Individual Skills Training Non Billable
Initial Evaluation
Telehealth—Evaluation and Management New Patient
Telehealth—Supervised Diagnostic Standard
Telehealth Comprehensive Evaluation
Telehealth Evaluation and Management Established Patient
Telehealth Initial Evaluation
Telehealth Pre Diagnostic Assessment
Telehealth Supervised Pre Diagnostic Assessment
Telehealth‐Diagnostic Assessment Standard
Phone Contact Non Billable
Pre Diagnostic Assessment
Program Development/School Paperwork
Reassessment
Referrals
Supervised Clinical Care Consultation, Phone
Supervised Comprehensive Evaluation
Supervised Diagnostic Extended
Supervised Initial Evaluation
Supervised Telehealth Comprehensive Evaluation
Supervised Telehealth Initial Evaluation
Supervised Pre Diagnostic Assessment
Supervised‐Diagnostic Assessment Standard
Supervised‐Family with Patient‐Telehealth
Treatment Interpretation
Treatment Plan Meeting
